# Workplace violence and turnover intention among the Bangladeshi female nurses after a year of pandemic: An exploratory cross-sectional study

**DOI:** 10.1371/journal.pgph.0000187

**Published:** 2022-04-01

**Authors:** Humayun Kabir, Saifur Rahman Chowdhury, Tajrin Tahrin Tonmon, Anjan Kumar Roy, Shimpi Akter, Mohammad Toyabur Rahaman Bhuya, Lukman Hossain, Samiul Amin Chowdhury, Shubrandu Sanjoy

**Affiliations:** 1 Department of Public Health, North South University, Dhaka, Bangladesh; 2 Department of Anthropology, Jahangirnagar University, Savar, Dhaka, Bangladesh; 3 Department of Nursing and Health Science, Jashore University of Science and Technology, Jashore, Bangladesh; 4 Department of Medical Studies, Bangladesh University of Professionals, Dhaka, Bangladesh; 5 School of Medical Sciences, Shahjalal University of Science & Technology, Sylhet, Bangladesh; 6 Department of Sociology, University of Dhaka, Dhaka, Bangladesh; 7 Department of Public Health, Leading University, Sylhet, Bangladesh; 8 Research Department, Saskatchewan Health Authority, Regina, Canada; University of Murcia, SPAIN

## Abstract

During the COVID-19 pandemic, workplace violence was widespread against healthcare personnel. Workplace violence (WPV) against nurses exhilarates their turnover intention (TI). The objective of this study was to investigate the association between workplace violence and turnover intention and also identify other factors associated with TI among Bangladeshi female nurses. An exploratory cross-sectional study was carried out among 881 female nurses between April 26 and July 10, 2021. The TI of the female nurses was the outcome variable of this study. The primary exposure variable was WPV faced by the nurses. Workplace Violence Scale (WPVS) was used to measure the WPV, and Turnover Intention Scale-6 (TIS-6) was used to measure the TI of the nurses. Multiple linear regression model was fitted to find the adjusted association of TI with WPV and other study variables. A stratified analysis by type of job (government vs. private) was also performed. The majority of the nurses (74.46%) faced low to high levels of WPV. The overall mean score of TIS was found 16.33 (± 4.72). Multiple linear regression analysis revealed that compared to government jobholders, the mean score of TIS (15.81 vs. 17.20) was found significantly higher among the private jobholders (p < 0.001). Nurses exposed to the intermediate and high level of WPV had a significantly higher TI score (β = 4.35, 95% CI: 3.36, 5.34) than the non-exposures. The TI of private jobholders was found significantly higher (β = 2.04, 95% CI: 1.09, 3.00) than the government jobholders. Compared to diploma degree holders, significantly higher TI was observed among the B.Sc. degree holders (β = 0.86, 95% CI: 0.22, 1.55) and M.Sc. degree holders (β = 1.46, 95% CI: 0.58, 2.34). Besides, the nurses who did not get timely salaries scored higher TI (β = 1.17, 95% CI: 0.12, 2.22). Moreover, the nurses who did not receive any training against WPV scored significantly higher TI (β = 1.89, 95% CI: 1.03, 2.74). The stratified analysis by type of job also revealed significant factors of TI in government and private settings. This study found a high prevalence of WPV and a high rate of TI among Bangladeshi female nurses. Moreover, this study explored an association between WPV and TI. The study findings could help policymakers facilitate a comfortable working environment by preventing WPV and addressing the factors to reduce nurses’ frequent TI.

## 1. Introduction

Turnover intention (TI) indicates that an employee aims to resign due to displeasure or work-related factors [1]. It is influenced by numerous factors, such as experience, education, organizational issues such as workplace violence (WPV), and organizational supports [2–4]. Therefore, nurses’ turnover is a challenging issue, which sometimes involves being exposed to frequent abusive behaviors [5]. Nurses’ turnover was reported to affect the quality of care and increased organization costs due to temporary replacement [6].

According to the World Health Organization, the incidents where the subjects are threatened, abused, or attacked explicitly while doing professional activities is defined as WPV [7]. Similarly, workplace aggression was reported as WPV in the medical system [8]. Evidence suggested that physical violence is also experienced by nurses frequently [9–11]. Certain studies indicated that healthcare workers are being harassed sexually at least once in their professional lives [12]. Shea et al. (2016) found that 67% of nurses experienced WPV over a year, whereas 20% experienced daily and 79% and 48% of those tortures were from patients and patients’ families, respectively [13]. Among the Bangladeshi health workers, 96% was physical violence, and 91% took place in public healthcare settings, whereas 61% of entry-level professionals were affected [14]. A study revealed that the quality of work-life among nurses in Bangladesh was unfavorable [15]. The caregiver personnel are exposed to a high risk of WPV, and nurses are the primary victims [16].

The rapid spread of COVID-19 cases has sparked a wave of violence against healthcare workers. Several incidents of violence, harassment, and stigmatization against healthcare employees, patients, and medical infrastructure have been reported in the aftermath of the COVID-19 pandemic; of these recorded events of violence and harassment, 67% were directed at healthcare personnel [17]. Besides, several studies reported that TI relatively increased among healthcare workers during the COVID-19 pandemic, and WPV had both direct and indirect effects on TI [18–20]. Moreover, during the COVID-19 pandemic, a considerable number of healthcare workers suffered negative mental health consequences. According to a recent study done among nurses in Bangladesh to evaluate mental health symptoms during the COVID-19 pandemic reported that the prevalence of mild to extremely severe depression was 50.5%, anxiety was 51.8%, and stress was 41.7% [21].

Reducing violence at the workplace led most nurses to retain [19]. Albeit, hostile environments accelerate turnover [22, 23]. A review identified that turnover rates varied among nurses in New Zealand (44.3%), the United States (26.8%), Canada (19.9%), and Australia (15.1%) [24]. Moreover, studies in China and Korea indicated that nurses at vulnerable WPV risk had elevated TI [22, 25]. WPV was reported to affect nurses mentally, physically, and exhaustion results in TI [26].

Factors should be addressed to take preventive measures against TI to maintain the workplace quality for nurses. Numerous studies investigated nurses’ TI and the influencing factors [6, 15, 27]. Similarly, studies specifically observed the role of WPV on nurses’ TI in different countries [28, 29]. However, studies on a small scale were conducted in Bangladesh concerning the physicians’ TI [30, 31]. To our best knowledge, no study has yet been conducted investigating WPV and TI among Bangladeshi nurses, specifically among females. Because female healthcare workers are more prone to WPV than male healthcare workers [32].

Therefore, this study aimed to investigate the role of WPV on female nurses’ TI in the context of a developing country like Bangladesh and address associated factors after a years of the COVID-19 pandemic. A prior study reported that workplace harassment and nurses’ well-being were highly varied by public vs. private sector in Bangladesh [33]. Thus, our study also aimed to specify the factors of TI by public sector vs. private sector stratification. The study findings could facilitate a safer and more flexible workplace for female nurses.

## 2. Materials and methods

### 2.1. Study design

An exploratory cross-sectional study was conducted among Bangladeshi registered female nurses between April 26 and July 10, 2021.

### 2.2. Study participants

A total of 881 female nurses participated in this study. The participants were directly involved with clinical care settings during the COVID-19 pandemic. Our calculated sample size was 784 at 80% power, 95% CI of 0.05 to 1.96, and 3.5% margin of error with an assumption of 50% of the nurses intended to turnover. The authors targeted to mitigate the margin of errors by obtaining more sample size than required. Therefore, additional 97 responses (11% of the calculated sample size) were also included in the final analysis.

### 2.3. Data collection procedure

A face-to-face interview was restricted due to COVID-19. Therefore, following convenience sampling method, a semi-structured self-reported questionnaire was distributed to collect data. The investigators followed online and offline data collection approaches. For online, participants were invited by an online questionnaire link (using "Google Form") that was distributed on different social media (Facebook, WhatsApp, etc.). By clicking on the link, willing nurses participated in the research. By online method of data collection, 490 completed responses were obtained. To achieve the required sample size, the authors distributed another 450 printed questionnaires in eight hospitals of two geographical divisions of Bangladesh by following the COVID-19 protocol. After one week, receiving 410 returned copies, 391 were obtained as completed responses.

The study objectives and responding procedure were described on the front page of the questionnaire. The demographic and work-related items were presented on the second page. The Workplace Violence Scale (WPVS) and Turnover Intention Scale (TIS-6) were subsequently presented on the third and fourth pages.

### 2.4. Study variables

The Turnover Intention (TI) of the female nurses was the outcome variable, and the exposure variable was WPV faced by the nurses. The socio-demographic variables (age, residence, geographical division of workplace, educational degree, and marital status) and work-related variables (type of job, hospital level, monthly salary, working experience, weekly working hours, department of work, timely salary, had sufficient equipment to work, had rewards for good work, got time for taking rest, average daily sleeping hours, and had training against WPV) were included.

### 2.5. Workplace violence measure

Five items Workplace Violence Scale (WPVS) was used to measure the violence perpetrated against the nurses by patients or their relatives [34, 35]. This scale was used previously among the nurses to conduct similar research [25, 36, 37]. The items were composed of physical assault (being spitted, bitten, pushed), emotional abuse (disrespect, negative attitude, cursing), threats (verbal, written, or physical threats to harm), verbal sexual harassment (comments, remarks, or questions of a sexual nature), and sexual abuse (any physical, sexual behaviors or unwanted touching). The responses indicated the frequencies (0 to 3 times) of WPV during the last 12 months. Total scores that ranged from 0 to 15 were calculated by summing all the responses of each item. The score 0 stands for WPV exposure status none, 1 to 5 for low, 6 to 10 for intermediate, and 11 to 15 for high level of WPV. The McDonald’s omega of WPVS was computed to assess the reliability of the scale among the nurse community in Bangladesh. The procedure yielded an omega of 0.60, an acceptable overall internal consistency of the tool.

### 2.6. Turnover intention measure

Turnover Intention Scale-6 (TIS-6) was used to measure the TI of the nurses during the past nine months [38, 39]. This six items scale was used among nurses by many studies [40, 41]. The responses of the items were on a five-point Likert scale. To respond to the TIS-6, participants needed to answer the questions which are concerned with their levels of turnover intention caused by their current job, their frequencies of seeking alternative jobs, and their extent of hesitation about leaving the current job by choosing a number from one to five. After summing all responses, the possible score ranges from 6 to 30. A higher score indicated a higher level of TI. The reliability coefficient, McDonald’s omega, was found 0.67.

### 2.7. Questionnaire development

In the questionnaire, both the original English version and Bengali translated version of the scales were used for a better understanding of the respondents. The questionnaire was translated from English into Bengali by two independent translators. The translated questionnaire was compared to the original English version by the authors. Some ambiguities were discussed with translators to prepare the Bengali version of the questionnaire. Face validation of the questionnaire was performed. Then, the questionnaire was distributed among five nurse-superintendents and five psychologists, and comments and suggestions regarding wording and the layout were received. Based on the suggestions, the wordings, meanings, and content of each item of the questionnaire were slightly modified. The refined questionnaire was then pilot tested among 20 nurses.

### 2.8. Statistical analysis

Descriptive statistics include mean, standard deviation (SD), frequency distribution, and percentage were performed for studied variables. Chi-square test and t-test were applied to show the differences of different level of WPV and mean score of TI between government job holders and private job holders, respectively. Linear regression was applied to determine the crude association of TI with WPV and other study variables. A multiple linear regression model was fitted to find an adjusted association of TI with WPV and other study variables. Subsequently, a hierarchical linear regression model was used to investigate the contributory role of studied factors on TI. Stratified analysis was also conducted by type of job (government vs. private). The p-value < 0.05 was considered statistically significant at a 95% confident interval. Data were analyzed by using statistical software STATA-16.

### 2.9. Ethical consideration

Ethical approval was obtained from the Ethical Review Board of Begum Rabeya Khatun Chowdhury Nursing College, Bangladesh. The approval ID is BRKCNC-IRB-2021/5. The ethics board approved implied consent through filling out the questionnaire. Freedom of refusal, withdrawing any time, the confidentiality of data, and briefing about study aim and objectives were ensured. As the questionnaire was self-reported, the participation of respondents in the study defined their implied consents.

## 3. Results

### 3.1. Descriptive statistics of the studied variables (n = 881)

The descriptive statistics of the studied variables are presented in **Table 1**. In total, 881 female nurses were included in the analysis of this study. The mean age of them was 28.69 (± 5.77) years. The majority of them (n = 762) were resided in the urban region (86.49%). In terms of educational degrees, diploma degree holders were 43.93%. Among the nurses, government job holders (n = 553) were 62.77%. More than two-thirds of the nurses (n = 645) worked at tertiary level hospitals (73.21%). Nearly half of them (46.42%) monthly salary was between 20,000 BDT to 29,999 BDT. Almost half of them (49.03%) weekly working hours were 36 hours or less. The nurses who did not get their salary on time was 9.42%. Nearly half of them (47.33%) did not have all the required equipment (n = 455) to manage patients properly. More than half of their (n = 555) average sleeping time was less than 8 hours (63.00%) per day. Lastly, 83.31% of nurses did not receive any training against WPV.

**Table 1:** Descriptive statistics of the studied variables (n = 881).

Sample characteristics	Sample distribution (n = frequency)	Percentage (%) / Mean (SD)
Demographic variables		
**Mean Age (years)**	881	28.69 (5.77)
**Age (years)**		
< 25	213	24.18
25–29	398	45.18
> 29	270	30.65
**Residence of the nurses**		
Rural	119	13.51
Urban	762	86.49
**Geographical division of workplace**		
Dhaka	450	51.08
Chattogram	81	9.27
Sylhet	255	28.94
Others	95	10.78
**Educational degree**		
M.Sc.	145	16.46
B.Sc.	349	39.61
Diploma	387	43.93
**Marital status**		
Unmarried	384	43.59
Married	497	56.41
**Work-related variables**		
**Type of job**		
Government	553	62.77
Private	328	37.23
**Hospital level**		
Tertiary	645	73.21
Secondary	133	15.10
Primary	103	11.69
**Monthly salary (BDT)**		
< 20,000	198	22.47
20,000–29,999	409	46.42
≥ 30,000	274	31.10
**Working experience**		
< 3 years	297	33.71
3–5 years	261	29.63
≥ 6 years	323	36.66
**Weekly working hours**		
≤ 36 hours	431	49.03
37–48 hours	348	39.59
> 48 hours	100	11.38
**Department**		
Medicine	196	22.25
Critical	217	24.63
Surgery	159	18.05
Gynecology	93	10.56
Emergency	28	3.18
COVID-19	34	3.86
Pediatric	40	4.54
General	114	12.94
**Timely salary**		
Yes	798	90.58
No	83	9.42
**Had sufficient equipment to work**		
Yes	464	52.67
No	417	47.33
**Had rewards for good work**		
Yes	196	22.25
No	685	77.75
**Got time for taking rest during working**		
Yes	467	53.01
No	414	46.99
**Average daily sleeping hours**		
≤ 7 hours	555	63.00
> 7 hours	326	37.00
**Had training against WPV**		
Yes	147	16.69
No	734	83.31

### 3.2. Distributions of the WPVS and TIS-6 score (n = 881)

**Fig 1:**
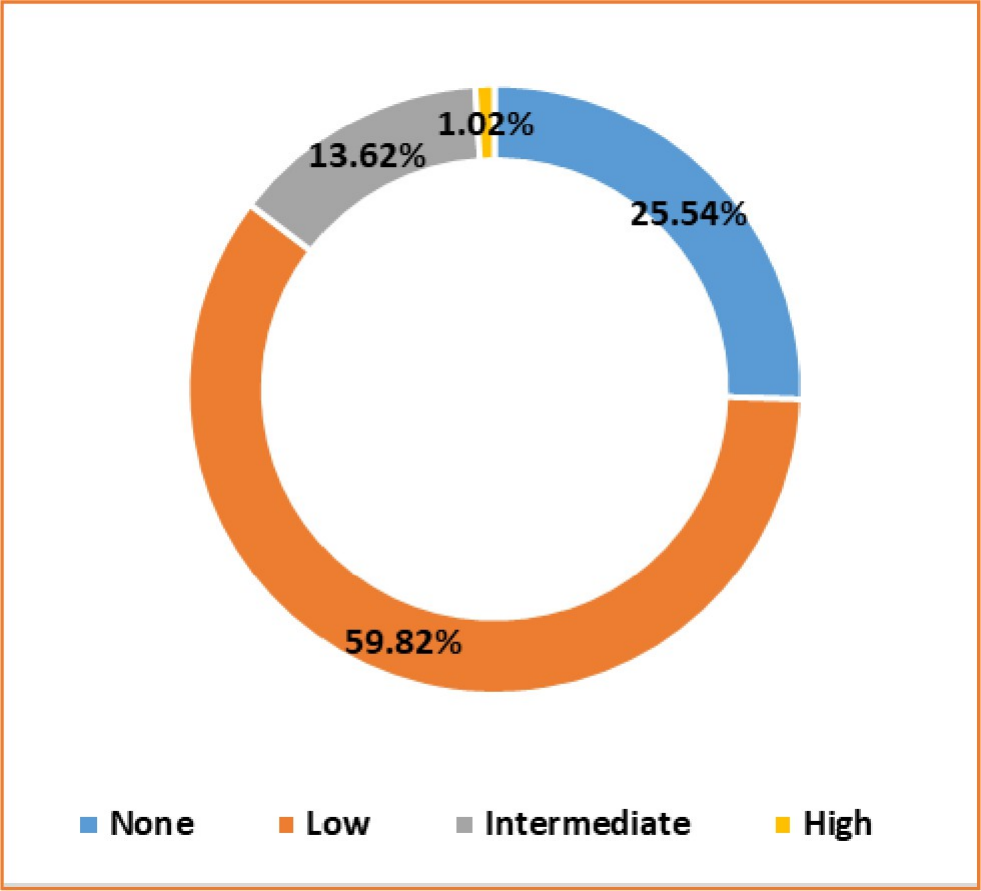
Prevalence of WPV among the female nurses (n = 881).

In **Table 2,** the level of workplace violence (WPV) among nurses and their Turnover Intention Scale (TIS) scores are presented. Approximately three-fourths of them were exposed to any degree of WPV (**Fig 1**). Among the nurses, 59.82% were exposed to a low level of WPV. Intermediate and high levels of WPV exposure were 13.62% and 1.02%, respectively. There was a significant difference in the degree of WPV between government and private employees (*p* = 0.001). The overall mean score of TIS was found 16.33 (± 4.72). Compared to government jobholders, the mean score of TIS (15.81 vs. 17.20) was found significantly higher among the private jobholders (*p* < 0.001).

**Table 2:** Level of workplace violence among nurses and TIS scores (n = 881).

Characteristics	Overall		Government job		Private job		*p*-value^†^
Variables	N	% / M±SD	N	% / M±SD	N	% / M±SD	
**Level of WPV**							**0.001**
None	225	25.54	119	21.52	106	32.32	
Low	527	59.82	341	61.66	186	56.71	
Intermediate	120	13.62	87	15.73	33	10.06	
High	9	1.02	6	1.08	3	0.91	
**TIS scores**	881	16.33 (4.72)	553	15.81 (4.64)	328	17.20 (4.73)	**< 0.001**

Note

^†^ *p-value estimated from chi-square test and t-test*.

### 3.3. Association of turnover intention with workplace violence and other study variables among the total participants

The adjusted and unadjusted association of TI with WPV and other study variables identified from the linear regression model are presented in **Table 3**. Compared to WPV non-exposure group, the TI score was found significantly higher (β = 2.24, 95% CI: 1.55, 2.93) among the low-level WPV exposure group. Similarly, nurses exposed to the intermediate and high level of WPV had a significantly higher TI score (β = 4.35, 95% CI: 3.36, 5.34) than the non-exposure. Compared to diploma degree holders, significantly higher TI was observed among the B.Sc. degree holders (β = 0.86, 95% CI: 0.22, 1.55) and M.Sc. degree holders (β = 1.46, 95% CI: 0.58, 2.34). The TI of private jobholders was found significantly higher (β = 2.04, 95% CI: 1.09, 3.00) than the government jobholders. Besides, in the adjusted analysis, the nurses who did not get timely salaries scored higher TI (β = 1.17, 95% CI: 0.12, 2.22) than those who got their salary on time. Moreover, the nurses who did not receive any training against WPV scored significantly higher (β = 1.89, 95% CI: 1.03, 2.74) than those who received any training against WPV. There were no significant differences in TI between nurses who had and who did not have the arrangement of taking rest during their working hours.

**Table 3:** Linear regression model to find the association of turnover intention with workplace violence and other study variables among the total participants (n = 881).

Variables	Unadjusted *β* (95% CI)	*p*-value	Adjusted *β* (95% CI)	*p*-value
**WPV**				
None	Reference		Reference	
Low	2.23 (1.53, 2.95)	**< 0.001**	2.24 (1.55, 2.93)	**< 0.001**
Intermediate & high	4.35 (3.37, 5.33)	**< 0.001**	4.35 (3.36, 5.34)	**< 0.001**
**Age (years)**				
< 25	Reference		References	
25–29	-0.66 (-1.44, 0.13)	0.100	0.04 (-0.88, 0.95)	0.937
> 29	-1.18 (-2.03, -0.34)	**0.006**	0.18 (-1.13, 1.49)	0.789
**Residence of the nurses**				
Rural	Reference		Reference	
Urban	0.27 (-0.65, 1.18)	0.567	-0.001 (-0.90, 0.89)	0.994
**Geographical division of workplace**				
Dhaka	Reference		Reference	
Chattogram	-1.34 (-2.44, -0.23)	**0.018**	-1.09 (-2.27, 0.09)	0.070
Sylhet	1.10 (0.28, 1.71)	**0.007**	0.86 (0.12, 1.60)	**0.022**
Others	-0.54 (-1.58, 0.50)	0.306	-0.88 (-1.94, 0.17)	0.102
**Educational degree**				
M.Sc.	0.78 (-0.12, 1.68)	0.091	1.46 (0.58, 2.34)	**0.001**
B.Sc.	0.85 (0.17, 1.54)	**0.014**	0.86 (0.22, 1.55)	**0.009**
Diploma	Reference		Reference	
**Marital status**				
Unmarried	0.75 (0.12, 1.38)	**0.019**	0.38 (-0.31, 1.07)	0.282
Married	Reference		Reference	
**Type of Job**				
Government	Reference		Reference	
Private	1.39 (0.75, 2.03)	**< 0.001**	2.04 (1.09, 3.00)	**< 0.001**
**Hospital level**				
Tertiary	0.64 (-0.34, 1.62)	0.201	0.59 (-0.55, 1.73)	0.308
Secondary	0.18 (-1.04, 1.40)	0.771	-0.30 (-1.51, 0.92)	0.633
Primary	Reference		Reference	
**Monthly salary (BDT)**				
< 20,000	Reference		Reference	
20,000–29,999	-1.53 (-2.32, -0.74)	**< 0.001**	-0.16 (-1.14, 0.81)	0.742
≥ 30,000	-1.72 (-2.57, -0.86)	**< 0.001**	-0.19 (-1.33, 0.95)	0.740
**Working experience**				
< 3 years	1.34 (0.60, 2.08)	**< 0.001**	0.76 (-0.29, 1.81)	0.153
3–5 years	0.43 (-0.34, 1.19)	0.273	0.21 (-0.72, 1.14)	0.653
≥ 6 years	Reference		Reference	
**Weekly working hours**				
≤ 36 hours	Reference		Reference	
37–48 hours	0.23 (-0.44, 0.90)	0.500	-0.17 (-0.83, 0.49)	0.610
> 48 hours	0.45 (-0.58, 1.48)	0.394	-0.51 (-1.51, 0.53)	0.338
**Department**				
Medicine	Reference		Reference	
Critical	-0.17 (-1.08, 0.74)	0.715	-0.20 (-1.07, 0.66)	0.643
Surgery	0.24 (-0.75, 1.23)	0.633	0.79 (-0.15, 1.73)	0.100
Gynecology	1.34 (0.18, 2.51)	**0.024**	1.22 (0.13, 2.31)	**0.029**
Emergency	0.77 (-1.10, 2.64)	0.419	0.72 (-1.07, 2.52)	0.429
COVID-19	0.33 (-1.39, 2.04)	0.711	0.66 (-0.94, 2.26)	0.420
Pediatric	0.49 (-1.12, 2.09)	0.551	1.09 (-0.40, 2.58)	0.153
General	0.33 (-0.77, 1.42)	0.558	0.90 (-0.13, 1.93)	0.086
**Timely salary**				
Yes	Reference		Reference	
No	2.71 (1.65, 3.76)	**< 0.001**	1.17 (0.12, 2.22)	**0.029**
**Had sufficient equipment to work**				
Yes	Reference		Reference	
No	0.82 (0.20, 1.44)	**0.010**	0.31 (-0.34, 0.96)	0.346
**Had rewards for good work**				
Yes	Reference		Reference	
No	1.94 (1.20, 2.68)	**< 0.001**	0.75 (-0.02, 1.52)	0.055
**Got time for taking rest during working**				
Yes	Reference		Reference	
No	0.54 (-0.09, 1.16)	0.092	0.09 (-0.53, 0.71)	0.768
**Average daily sleeping hours**				
≤ 7 hours	0.48 (-0.16, 1.13)	0.143	0.36 (-0.25, 0.97)	0.250
> 7 hours	Reference		Reference	
**Had training against WPV**				
Yes	Reference		Reference	
No	2.17 (1.35, 2.99)	**< 0.001**	1.89 (1.03, 2.74)	**< 0.001**

### 3.4. Job stratified analysis: Association of turnover intention with workplace violence and other study variables

#### 3.4.1. Turnover intention among the government employed female nurses

The adjusted and unadjusted association of TI with WPV and other study variables identified from the linear regression model stratified by type of job are presented in **Table 4.** The variables to predict TI among the female nurses were varied significantly by type of job. Compared to WPV non-exposure group, a significantly higher TI was observed among government jobholders exposed to a low level of WPV (*β* = 2.02, 95% CI: 1.12, 2.92) and intermediate and high level of WPV (*β* = 4.38, 95% CI: 3.17, 5.59). The TI score was found significantly higher among the M.Sc. (*β* = 1.52, 95% CI: 0.54, 2.49) and B.Sc. (*β* = 1.02, 95% CI: 0.17, 1.88) degree holders than the diploma degree holders government-employed nurses. Similarly, the TI score was found significantly higher (β = 2.18, 95% CI: 0.36, 4.00) among the nurses of tertiary level hospitals than the nurses of primary level hospitals. Moreover, the TI was found significantly higher (*β* = 2.75, 95% CI: 0.79, 4.71) among those who did not get their salary timely. Similarly, the TI score was significantly higher (*β* = 1.85, 95% CI: 0.54, 3.15) among nurses who did not receive any training against WPV.

**Table 4:** Linear regression model to find the association of turnover intention with workplace violence and other study variables stratified by type of job (n = 881).

Variables	Government job				Private job			
	Unadjusted β (95% CI)	*p*-value	Adjusted β (95% CI)	*p*-value	Unadjusted β (95% CI)	*p*-value	Adjusted β (95% CI)	*p*-value
**WPV**								
None	Reference		Reference		Reference		Reference	
Low	2.44 (1.51, 3.36)	**< 0.001**	2.02 (1.12, 2.92)	**< 0.001**	2.46 (1.38, 3.53)	**< 0.001**	1.90 (0.81, 2.98)	**0.001**
Intermediate & high	4.60 (3.40, 5.80)	**< 0.001**	4.38 (3.17, 5.59)	**< 0.001**	4.89 (3.19, 6.60)	**< 0.001**	3.71 (1.94, 5.47)	**< 0.001**
**Age (years)**								
< 25	Reference				Reference		Reference	
25–29	1.80 (0.14, 3.46)	**0.033**	1.26 (-0.47, 3.00)	0.154	-0.64 (-1.67, 0.41)	0.229	-0.40 (-1.57, 0.78)	0.509
> 29	1.57 (-0.09, 3.22)	0.064	1.60 (-0.32, 3.53)	0.102	-1.51 (-5.10, 2.07)	0.407	-2.32 (-6.19, 1.56)	0.241
**Residence of the nurses**								
Rural	Reference		Reference		Reference		Reference	
Urban	0.59 (-0.60, 1.77)	0.332	-0.47 (-1.73, 0.79)	0.463	0.08 (-1.33, 1.49)	0.908	0.10 (-1.27, 1.47)	0.887
**Geographical division of workplace**								
Dhaka	Reference		Reference		Reference		Reference	
Chattogram	-1.64 (-3.10, -0.18)	**0.028**	-0.56 (-2.12, 1.00)	0.480	-1.39 (-3.07, 0.30)	0.108	-0.45 (-2.49, 1.59)	0.666
Sylhet	1.05 (0.15, 1.96)	**0.023**	1.25 (0.26, 2.25)	**0.013**	0.60 (-0.57, 1.78)	0.311	0.62 (-0.66, 1.90)	0.338
Others	-1.46 (-2.72, -0.18)	**0.024**	-1.44 (-2.82, -0.06)	**0.041**	0.87 (-0.87, 2.62)	0.325	0.38 (-1.33, 2.09)	0.663
**Educational degree**								
M.Sc.	1.52 (0.49, 2.55)	**0.004**	1.52 (0.54, 2.49)	**0.002**	1.28 (-1.07, 3.63)	0.284	1.64 (-0.62, 3.89)	0.155
B.Sc.	1.25 (0.37, 2.13)	**0.006**	1.02 (0.17, 1.88)	**0.019**	1.01 (-0.07, 2.10)	0.068	1.83 (-0.27, 1.92)	0.139
Diploma	Reference		Reference		Reference		Reference	
**Marital status**								
Unmarried	0.34 (-0.51, 1.20)	0.433	0.55 (-0.35, 1.44)	0.233	0.16 (-0.95, 1.26)	0.778	Reference	
Married	Reference		Reference		Reference		-0.10 (-1.19, 0.99)	0.852
**Hospital level**								
Tertiary	2.17 (0.70, 3.64)	**0.004**	2.18 (0.36, 4.00)	**0.019**	0.15 (-1.21, 1.51)	0.829	-0.47 (2.02, 1.08)	0.551
Secondary	0.34 (-1.49, 2.17)	0.716	0.28 (-1.63, 2.20)	0.771	0.30 (-1.30, 1.91)	0.711	-0.10 (-1.74, 1.54)	0.906
Primary	Reference		Reference		Reference		Reference	
**Monthly salary (BDT)**								
< 20,000	Reference		Reference		Reference		Reference	
20,000–29,999	1.49 (-1.31, 4.28)	0.297	-0.91 (-3.62, 1.81)	0.513	-0.59 (-1.71, 0.51)	0.302	0.08 (-1.16, 1.33)	0.896
≥ 30,000	1.90 (-0.91, 4.71)	0.185	-0.93 (-3.79, 1.94)	0.525	-3.26 (-4.87, -1.66)	**< 0.001**	-1.86 (-3.70, -0.02)	**0.047**
**Working experience**								
< 3 years	0.06 (-1.05, 1.18)	0.911	0.35 (-0.97, 1.66)	0.604	-0.58 (-2.59, 1.44)	0.574	-0.81 (-2.98, 1.35)	0.460
3–5 years	0.80 (-0.7, 1.68)	0.072	0.90 (-0.13, 1.93)	0.087	-2.41 (-4.55, -0.27)	0.027	-2.25 (-4.44, -0.06)	**0.044**
≥ 6 years	Reference		Reference		Reference		Reference	
**Weekly working hours**								
≤ 36 hours	Reference		Reference		Reference		Reference	
37–48 hours	-0.04 (-0.86, 0.79)	0.930	-0.24 (-1.07, 0.59)	0.567	0.02 (-1.17, 1.21)	0.976	-0.06 (-1.18, 1.07)	0.921
> 48 hours	-1.62 (-3.35, 0.12)	0.068	-1.69 (-3.40, 0.02)	0.053	0.56 (-0.89, 2.01)	0.444	0.12 (-1.32, 1.56)	0.866
**Department**								
Medicine	Reference		Reference		Reference		Reference	
Critical	-1.62 (-2.76, -0.48)	**0.006**	-1.52 (-2.62, -0.43)	**0.006**	1.79 (0.31, 3.27)	**0.018**	2.15 (0.75, 3.56)	**0.003**
Surgery	-0.41 (-1.51, 0.70)	0.471	0.16 (-0.94, 1.25)	0.777	2.79 (0.65, 4.93)	**0.011**	2.02 (-0.02, 4.05)	0.052
Gynecology	0.79 (-0.75, 2.34)	0.314	1.10 (-0.36, 2.55)	0.139	2.17 (0.40, 3.94)	**0.016**	1.89 (0.15, 3.63)	**0.033**
Emergency	1.31 (-1.02, 3.63)	0.269	0.40 (-1.87, 2.66)	0.730	0.08 (-2.92, 3.08)	0.958	0.86 (-2.21, 3.94)	0.582
COVID-19	-1.82 (-3.86, 0.22)	0.081	-1.10 (-3.03, 0.82)	0.260	4.81 (1.81, 7.81)	**0.002**	4.12 (1.23, 7.01)	**0.005**
Pediatric	-0.46 (-2.43, 1.51)	0.647	0.45 (-1.41, 2.31)	0.633	2.18 (-0.46, 4.81)	0.105	2.49 (-0.05, 5.03)	0.055
General	-0.87 (-2.29, 0.55)	0.228	0.38 (-0.98, 1.75)	0.584	1.84 (0.15, 3.52)	**0.033**	2.39 (0.76, 4.02)	**0.004**
**Timely salary**								
Yes	Reference		Reference		Reference			
No	2.84 (0.78, 4.90)	**0.007**	2.75 (0.79, 4.71)	**0.006**	2.05 (0.76, 3.33)	**0.002**	0.41 (-0.88, 1.69)	0.534
**Had sufficient equipment to work**								
Yes	Reference		0.02 (-0.78, 0.83)	0.954	Reference		Reference	
No	1.02 (0.21, 1.83)	**0.014**	Reference		2.08 (1.10, 3.16)	**< 0.001**	1.27 (0.15, 3.00)	**0.027**
**Had rewards for good work**								
Yes	Reference		0.85 (-0.07, 1.76)	0.069	Reference		0.33 (-1.11, 1.76)	0.654
No	1.46 (0.57, 2.35)	**0.001**	Reference		2.61 (1.32, 3.90)	**< 0.001**	Reference	
**Got time for taking rest during working**								
Yes	Reference		Reference		Reference		Reference	
No	0.30 (-0.48, 1.08)	0.444	0.30 (-0.52, 1.11)	0.474	0.66 (-0.37, 1.69)	0.205	-0.14 (-1.15, 0.88)	0.792
**Average daily sleeping hours**								
≤ 7 hours	-0.05 (-0.85, 0.76)	0.907	0.23 (-0.57, 1.03)	0.575	1.37 (0.32, 2.43)	**0.011**	1.05 (0.03, 2.07)	**0.045**
> 7 hours	Reference		Reference		Reference		Reference	
**Had training against WPV**								
Yes	Reference		Reference		Reference		Reference	
No	3.25 (1.96, 4.55)	**< 0.001**	1.85 (0.54, 3.15)	**0.006**	2.50 (1.39, 3.60)	**< 0.001**	1.45 (0.25, 2.66)	**0.018**

#### 3.4.2. Turnover intention among the privately employed female nurses

In the private sector, compared to non-exposed nurses to WPV, TI was found significantly higher among the exposed nurses to low level of WPV (*β* = 1.90, 95% CI: 0.81, 2.98) and intermediate and high level of WPV (*β* = 3.71, 95% CI: 1.94, 5.47). Among the highest-paid (≥ 30,000 BDT per month) nurses, the TI was found significantly lower (*β* = -1.86, 95% CI: -3.70, -0.02), compared to the lowest paid nurses (< 20,000 BDT per month). Compared to the highest working experienced nurses (≥ 6 years), the TI of moderately experienced nurses (3 to 5 years) were found significantly lower (*β* = -2.25, 95% CI: -4.44, -0.06). Compared to the nurses of medicine ward, TI score was found significantly higher among the nurses of critical care (*β* = 2.15, 95% CI: 0.74, 3.56), gynecological (*β* = 1.89, 95% CI: 0.15, 3.63), COVID-19 (*β* = 4.12, 95% CI: 1.23, 7.01), and general ward (*β* = 2.39, 95% CI: 0.76, 4.02). Intended to turnover was found significantly higher (*β* = 1.05, 95% CI: 0.03, 2.07) among those who had lower average sleeping hours per day (≤ 7 hours) compared to the nurses of higher sleeping hours (> 7 hours). Lastly, among the privately employed nurses, non-trained against WPV were significantly more intended to turnover (*β* = 1.45, 95% CI: 0.25, 2.66).

### 3.5. Predictive models of turnover intention among the total participants

The predictive models of TI among the total participants are presented in **Table 5**. The demographic and work-related variables explained 2.4% and 10.0% of TI variance (in **block 1** and **block 2**), respectively. In **block 3**, the addition of WPV contributed to a variance of 7.0%, and the overall explanatory variance jumped to 19.4%.

**Table 5:** Predictive models of turnover intention (n = 881).

Variables	Block-1 (β)	Block-2 (β)	Block-3 (β)
Age	-0.67**	0.16	0.29
Residence of the nurses	-0.37	-0.17	-0.02
Geographical division of workplace	0.19	0.08	0.08
Educational degree	-0.74**	-0.75**	-0.68**
Marital status	0.42	0.34	0.37
Type of job		1.82***	2.30***
Hospital level		-0.71**	-0.77**
Monthly salary		-0.26	-0.19
Working experience		-0.24	-0.33
Weekly working hours		-0.01	-0.19
Department		0.03	0.05
Timely salary		1.80**	1.12*
Had sufficient equipment to work		0.66*	0.22
Had rewards for good work		1.03*	0.78*
Got time for taking rest during working		0.14	-0.01
Average daily sleeping hours		-0.36	-0.31
Had training against WPV		2.10***	2.06***
WPV			-2.04***
F	4.22***	7.15***	11.50***
R^2^	0.024	0.124	0.194
Δ R^2^		0.100***	0.070***
Adjusted R^2^	0.018	0.106	0.177

Note

*p < 0.05

**p < 0.01

***p < 0.001.

### 3.6. Predictive models of turnover intention by type of job

The predictive models of TI by type of job are presented in **Table 6**. Among the government jobholders, the demographic and work-related variables explained 2.2% and 10.8% of the variance of TI (in **block 1** and **block 2**), respectively. Adding WPV in the final model (in **block 3**), the explanatory variance of TI jumped by 7.1%, and the overall variance was fixed at 20.1%. On the other hand, among the private jobholders, the demographic and work-related variables explained 2.8% and 14.1% variance of TI (in **block 1** and **block 2**), respectively. In the final model, WPV contributed to an additional 5.4% (in **block 3**) to fix the total predictability by 22.22% of TI among the privately employed nurses.

**Table 6:** Predictive models of turnover intention by job type (n = 881).

Variables	Block-1 (*β*)		Block-2 (*β*)		Block-3 (*β*)	
	Govt.	Private	Govt.	Private	Govt.	Private
Age	0.32	-0.97	0.39	-0.74	0.45	-0.48
Residence of the nurses	-0.68	-0.17	0.27	-0.05	0.68	-0.12
Geographical division of workplace	0.01	0.32	0.13	0.06	0.11	0.15
Educational degree	-0.78**	-1.04*	-0.74**	0.02	-0.64*	-1.04*
Marital status	0.56	0.06	0.64	0.07	0.50	0.21
Hospital level			-1.50***	-1.15**	-1.64***	-0.02
Monthly salary			0.28	-0.08	0.16	-0.90*
Working experience			-0.38	0.18	-0.34	-0.28
Weekly working hours			-0.16	-0.32*	-0.47	0.09
Department			0.16	1.11	0.14	-0.25*
Timely salary			3.46**	1.15*	2.69**	0.49
Had sufficient equipment to work			0.39	0.87	-0.17	0.88
Had rewards for good work			1.01*	-0.10	0.86	0.57
Got time for taking rest during working			0.43	-1.03	0.29	-0.19
Average daily sleeping hours			0.01	-1.03	0.05	-0.91
Had training against WPV			2.72***	1.52*	2.30**	1.71**
WPV					-2.04***	-1.83***
F	2.41*	1.85	4.98***	3.93***	7.89***	5.19***
R^2^	0.022	0.028	0.130	0.169	0.201	0.222
Δ R^2^			0.108***	0.141***	0.071***	0.054***
Adjusted R^2^	0.013	0.013	0.104	0.126	0.175	0.179

Note

*p < 0.05

**p < 0.01

***p < 0.001.

## 4. Discussion

Our study investigated turnover intention (TI) among the female nurses of Bangladesh and its associated factors, particularly the role of workplace violence (WPV) on TI. Our study showed that the mean score of TI is relatively high among the participant nurses. Studies conducted during the COVID-19 pandemic in China, Philippines, and Bolivia also reported a relatively high prevalence of TI among healthcare professionals [18, 20, 42]. The results showed that almost one-fourth of the nurses, faced any degree of WPV, and their TI score was significantly higher. This finding is consistent with the other studies conducted in different countries [2, 43]. This finding is also in line with research undertaken during the pandemic among healthcare professionals in China, where their TI was predicted by their level of WPV exposure [19]. In this study, WPV was significantly associated with higher TI in employees from both the government and private sectors, consistent with earlier studies [6, 9, 29]. Negligence towards the nurses could be a reason for experiencing higher WPV that may hinder their quality of life, increase turnover, and exude low-quality patient care [44]. Furthermore, throughout the pandemic, the rate of WPV exposure among healthcare personnel has grown dramatically [17]. On the other hand, having no training against WPV would cause a significant reason for fear and dissatisfaction; the employees could end up leaving their job. This study found that nurses without any training against WPV score higher TI compared to those been trained. Chang et al. (2018) reported that any consultation with the supervisors is cost-effective to keep the employees motivated in their job [45]. Therefore, effective managerial support, training, and leadership, as well as a fair and balanced distribution of facilities, are required to prevent WPV.

The TI was found as significantly higher among the nurses from the private healthcare facilities. In Bangladeshi private hospitals, the quality of care is better than the public hospitals, although nurses are highly loaded and lowly paid [46]. The unfavorable workplace could hinder the durability of the worker’s stay, which is found varied in accordance with the government job holder vs. private job holder [2, 47–49]. Therefore, ensuring quality care with comparatively lower payments or job insecurity could exile TI.

In this study, an association was found as significant between educational degrees and TI. Higher educated nurses scored higher TI. This finding may be explained by that in Bangladesh, higher education is less valued in positioning or promoting of the nurses. Several studies also reported that more professional and educational qualifications were significantly associated with high TI [15, 50–52]. In the government sector, nurses from tertiary level hospitals were more prone to turnover. Bangladeshi studies found that the healthcare workers were neglected and ill-treated, especially in tertiary-level hospitals [14, 15]. Similary, Andualem Wubetie et al. (2020) reported a possible explanation that the excessive number of patients being admitted in public settings [49]. In the private sector, monthly salary was addressed as to predict TI. This finding is correlated with a previous study conducted by Akter et al. (2018) that reported a lower salary structure for the Bangladeshi nurses that predicted the quality of working life [15]. Moreover, higher TI was observed among those not getting timely salaries. A study showed that higher job satisfaction was correlated with timely payment [6]. As Bangladesh is a developing country, payment time could exile nurses’ TI.

Increased working experience was observed to be associated with a reduced TI in private settings. Similarly, Kim et al. found that highly experienced nurses were less intent to leave [53]. One possible explanation could be that nurses’ salaries increased based on experience. Higher TI was found among the nurses of critical care unit in government settings. In private settings, nurses of critical care, COVID-19, gynecological, and general departments were higher intended to turnover. During the COVID-19 pandemic, the patients’ flow was mostly critical care oriented. The sudden patients’ flow with unready settings might attribute to their TI [41]. Lack of sufficient equipment to work was found to be a predictor of nurses’ higher TI in the private sector. The finding is consistent with other studies, reported that proper equipment supplies facilitated nurses’ working environment and deducted TI [9, 45]. Higher turnover was observed among the nurses who slept less than seven hours. However, this was found differently in other studies of Korea and Turkey [9, 54]. In government settings, nurses from the Sylhet division, the northeastern part of Bangladesh, were more prone to TI. The possible explanation could be that respondents’ degree of exposure to independent variables could vary in different geographical locations.

In this study, getting adequate time to take rest during working hours was not found to be associated with nurses’ TI significantly. On the contrary, Deng et al. reported that time for taking rest at working place was correlated with turnover [55].

### 4.1 Strengths and limitations

To our best knowledge, this is the only study that addressed the association between WPV and TI, and other associated factors of TI among Bangladeshi female nurses. Nurses from all geographical divisions of the country got opportunities to participate, which resulted in an optimal sample size in the study. Another strength is that this research only included female nurses as they are more vulnerable to WPV and TI in the perspective of Bangladesh. Thus, sex is not an effect modifier or confounder to the outcome of this study. However, this research has some limitations also. As a non-random sampling technique was applied, selection bias could not be excluded. In addition, the risk of information bias might be present due to the self-reported questionnaire. Finally, as a nature of a cross-sectional study, causality could not be established. Further in-depth and rigorous research is essential on nurses’ TI and WPV for establishing sustained work environments and increasing retention.

## 5. Conclusions

This study found a high prevalence of WPV and a high rate of TI among Bangladeshi female nurses. Moreover, this study explored an association between WPV and TI, and also identified some significant factors that predict TI of nurses. Nurses from private settings and the WPV exposed groups were more intended to turnover. This study findings might help policymakers facilitate a comfortable working environment by preventing WPV and addressing the factors to reduce nurses’ frequent TI.

## 6. Implications for nursing practice

During the COVID-19 pandemic, workplace violence was widespread against healthcare personnel that lead to their turnover intention. Therefore, hospital authorities can take proper initiatives based on study findings to reduce WPV for safeguarding nurses working environments to ensure workplace retention. Finally, the government may highlight nurses’ contributions in the clinical settings to get support from the general people.

## Supporting information

S1 File Name of the research assistants. (DOCX)

S1 Data Dataset of the study. (XLS)
