# Supplementary material for: Workplace violence and turnover intention among the Bangladeshi female nurses after a year of pandemic: An exploratory cross-sectional study
Source: PLOS Glob Public Health. 2022 Apr 1;2(4):e0000187. doi: 10.1371/journal.pgph.0000187 (PMC10021668; doi:10.1371/journal.pgph.0000187)
Supplement: S1 File — (DOCX) [file pgph.0000187.s001.docx]

**Most acknowledgeable Research Assistants**

| **Serial** | **Name** | **Affiliation** |
| --- | --- | --- |
|  | Md. Ikbal Hossain | Begum Rabeya Khatun Chowdhury Nursing College, Sylhet |
|  | Ahmmad Refaye | Dhaka Nursing College, Dhaka |
|  | Fahima Begum | Sylhet Nursing College, Sylhet |
|  | Md. Ajijur Rahman | Begum Rabeya Khatun Chowdhury Nursing College, Sylhet |
|  | Bubli Moni Dash | Begum Rabeya Khatun Chowdhury Nursing College, Sylhet |
|  | Rumi Begum | Begum Rabeya Khatun Chowdhury Nursing College, Sylhet |
|  | Jamil Ahmed | Sylhet Women's Nursing College, Sylhet |
|  | Shipa Akther | Begum Rabeya Khatun Chowdhury Nursing College, Sylhet |
|  | Kamrun Nahar Happy | Begum Rabeya Khatun Chowdhury Nursing College, Sylhet |
|  | Laboni Begum | Begum Rabeya Khatun Chowdhury Nursing College, Sylhet |
|  | Kanta Nath | Begum Rabeya Khatun Chowdhury Nursing College, Sylhet |
|  | Sumita Dhor | Begum Rabeya Khatun Chowdhury Nursing College, Sylhet |
|  | Tulika Debi | Begum Rabeya Khatun Chowdhury Nursing College, Sylhet |
|  | Sujan Bishwas | Begum Rabeya Khatun Chowdhury Nursing College, Sylhet |
|  | Khushbul Alam Rifat | Begum Rabeya Khatun Chowdhury Nursing College, Sylhet |
|  | Sharmi Paul | Begum Rabeya Khatun Chowdhury Nursing College, Sylhet |
|  | Md. Nurul Hoque | Begum Rabeya Khatun Chowdhury Nursing College, Sylhet |
|  | Farhana Akter Reba | Begum Rabeya Khatun Chowdhury Nursing College, Sylhet |
|  | Ashok Chandra Das | Begum Rabeya Khatun Chowdhury Nursing College, Sylhet |
|  | Md Saifur Rahman | Begum Rabeya Khatun Chowdhury Nursing College, Sylhet |
|  | Tahera Jannath Tanny | Begum Rabeya Khatun Chowdhury Nursing College, Sylhet |
|  | Taslima Begom | Begum Rabeya Khatun Chowdhury Nursing College, Sylhet |
